# *QuickStats:* Percentage of Births to Mothers Who Reported Smoking Cigarettes at Any Time During Pregnancy, by Urbanization Level* of County of Residence — United States, 2020

**DOI:** 10.15585/mmwr.mm7047a5

**Published:** 2021-11-26

**Figure:**
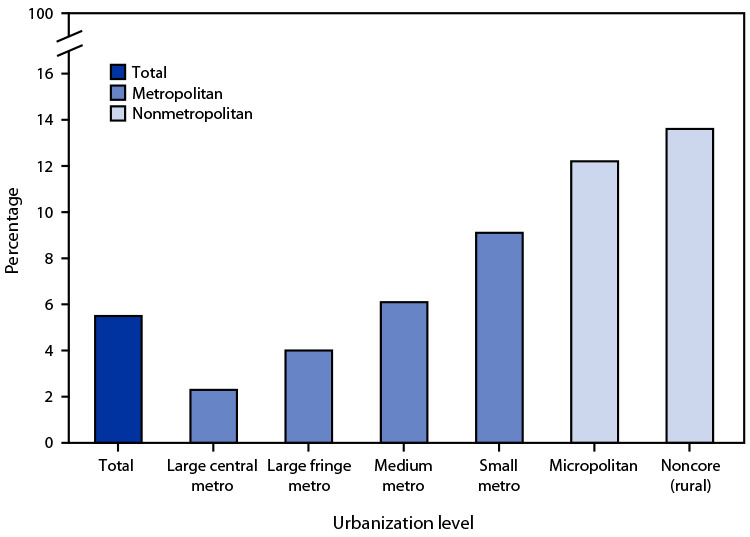
In 2020, 5.5% of all births were to women who reported smoking cigarettes at any time during pregnancy. This percentage was lowest in large central metropolitan areas (2.3%) and increased as the county of residence became less urbanized, reaching a high of 13.6% in the most rural (noncore) counties.

**Source:** National Vital Statistics System, natality file. https://wonder.cdc.gov/natality-expanded-current.html

